# Depressive and anxiety symptoms in adults during the COVID-19 pandemic in England: A panel data analysis over 2 years

**DOI:** 10.1371/journal.pmed.1004144

**Published:** 2023-04-18

**Authors:** Feifei Bu, Andrew Steptoe, Daisy Fancourt

**Affiliations:** Department of Behavioural Science and Health, University College London, London, United Kingdom; Addis Ababa University / King’s College London, ETHIOPIA

## Abstract

**Background:**

There has been much research into the mental health impact of the Coronavirus Disease 2019 (COVID-19) pandemic and how it is related to time-invariant individual characteristics. However, there is still a lack of research showing long-term trajectories of mental health across different stages of the pandemic. And little is known regarding the longitudinal association of time-varying factors with mental health outcomes. This study aimed to provide a longitudinal profile of how mental health in adults changed across different stages of the COVID-19 pandemic and to examine their longitudinal associations with time-varying contextual (e.g., COVID-19 policy response and pandemic intensity) and individual level factors.

**Methods and findings:**

This study used data from a large panel study of over 57,000 adults living in England, who were followed up regularly for 2 years between March 2020 and April 2022. Mental health outcomes were depressive and anxiety symptoms. Depressive symptoms were assessed by the Patient Health Questionnaire (PHQ-9) and anxiety symptoms by the Generalized Anxiety Disorder assessment (GAD-7). Entropy balancing weights were applied to restore sample representativeness. After weighting, approximately 50% of participants were female, 14% from ethnic minority backgrounds, with a mean age of 48 years. Descriptive analyses showed that mental health changes were largely in line with changes in COVID-19 policy response and pandemic intensity. Further, data were analysed using fixed-effects (FE) models, which controlled for all time-invariant confounders (observed or not). FE models were fitted separately across 3 stages of the COVID-19 pandemic, including the first national lockdown (21/03/2020–23/08/2020), second and third national lockdowns (21/09/2020–11/04/2021), and “freedom” period (12/04/2021–14/11/2021). We found that more stringent policy response (measured by stringency index) was associated with increased depressive symptoms, in particular, during lockdown periods (β = 0.23, 95% confidence interval (CI) = [0.18 to 0.28], *p* < 0.001; β = 0.30, 95% CI = [0.21 to 0.39], *p* < 0.001; β = 0.04, 95% CI = [−0.03 to 0.12], *p* = 0.262). Higher COVID-19 deaths were also associated with increased depressive symptoms, but this association weakened over time (β = 0.29, 95% CI = [0.25 to 0.32], *p* < 0.001; β = 0.09, 95% CI = [0.05 to 0.13], *p* < 0.001; β = −0.06, 95% CI = [−0.30 to 0.19], *p* = 0.655). Similar results were also found for anxiety symptoms, for example, stringency index (β = 0.17, 95% CI = [0.12 to 0.21], *p* < 0.001; β = 0.13, 95% CI = [0.06 to 0.21], *p* = 0.001; β = 0.10, 95% CI = [0.03 to 0.17], *p* = 0.005), COVID-19 deaths (β = 0.07, 95% CI = [0.04 to 0.10], *p* < 0.001; β = 0.04, 95% CI = [0.00 to 0.07], *p* = 0.03; β = 0.16, 95% CI = [−0.08 to 0.39], *p* = 0.192). Finally, there was also evidence for the longitudinal association of mental health with individual level factors, including confidence in government/healthcare/essentials, COVID-19 knowledge, COVID-19 stress, COVID-19 infection, and social support. However, it is worth noting that the magnitudes of these longitudinal associations were generally small. The main limitation of the study was its non-probability sample design.

**Conclusions:**

Our results provided empirical evidence on how changes in contextual and individual level factors were related to changes in depressive and anxiety symptoms. While some factors (e.g., confidence in healthcare, social support) clearly acted as consistent predictors of depressive and/or anxiety symptoms, other factors (e.g., stringency index, COVID-19 knowledge) were dependent on the specific situations occurring within society. This could provide important implications for policy making and for a better understanding of mental health of the general public during a national or global health crisis.

## Author summary

**Why was this study done?:**

- There is increasing research showing worsening mental health during the Coronavirus Disease 2019 (COVID-19) pandemic.
- There is some evidence for mental health changes across different stages of the pandemic, but there is a lack of long-term longitudinal research with regular follow-ups.
- Few studies have examined how changes in contextual and individual factors are related to mental health outcomes.

**What did the researchers do and find?:**

- Drawing data from over 57,000 participants, we showed descriptively how depressive and anxiety symptoms changed over time between March 2020 and April 2022.
- Using fixed-effects (FE) analyses, we found evidence for longitudinal associations of depressive and anxiety symptom with both contextual (e.g., stringency index, COVID-19 cases) and individual factors (e.g., COVID-19 knowledge, social support).
- Some of these longitudinal associations (e.g., stringency index, COVID-19 knowledge) differed across different stages of the COVID-19 pandemic, and differential relationships were also found between depressive and anxiety symptoms.

**What do these findings mean?:**

- These findings from observational data provide empirical evidence for the longitudinal association between contextual factors and people’s mental health, offering important implications for policy making.
- Our study highlights the importance of individual level factors such as confidence in healthcare services and perceived social support in supporting better mental health.
- Further research is needed to monitor mental health changes over longer periods and to use alternative mental health assessment tools.

## Introduction

The Coronavirus Disease 2019 (COVID-19) pandemic has caused unprecedented disruptions to the global economy and people’s everyday lives. It has presented various psychological challenges, such as uncertainty and fear about the virus, enforced social isolation caused by lockdown and social distancing measures, financial adversities, reduced access to healthcare services, and so forth [1,2]. At the start of the pandemic, this led to widespread concerns about what the global mental health impact would be [3,4], with subsequent empirical evidence showing worsening mental health during, compared to prior to, the pandemic [5–7]. However, in the 2 years since, the political and public health contexts have changed substantially with the roll out of vaccination and easing of social restrictions in many countries. Over this period, there has been some evidence from longitudinal studies for a gradual improvement in mental health from the start of the pandemic [2,8,9], followed by deteriorations during the second wave of COVID-19 [10,11]. But the follow-up periods of such studies have been relatively short to provide a long-term perspective of mental health changes across different stages of the COVID-19 pandemic. To the best of our knowledge, no study to date has shown detailed longitudinal changes in public mental health over the first 2 years of the pandemic.

Further, numerous studies have shown how mental health during the pandemic is related to sociodemographic factors (e.g., age, gender, socioeconomic position, health conditions) [2,7], most of which are time invariant, focusing on individual characteristics. There has been a much smaller literature on the relationship of time-varying and, in particular, contextual factors with mental health, such as COVID-19 cases, deaths, and policy responses. A meta-analysis of depression outcomes across 33 countries found that the prevalence of clinical depression was significantly lower in countries where stringent policies were implemented in early 2020 [12]. This is consistent with another cross-country study showing that overall stringency was associated with lower Google searches for “depression,” but no evidence for “anxiety” in 2020 [13]. However, a recent study using longitudinal data between April 2020 and June 2021 from 15 countries showed that higher policy stringency and number of deaths were both associated with poor mental health outcomes [14]. These inconsistencies could reflect the differential association of mental health with contextual factors across different stages of the COVID-19 pandemic and potential differences between depression and anxiety. Further, it is relevant to consider whether predictors of mental health during the pandemic have changed as the context of COVID-19 has shifted. Indeed, research into behaviours during the pandemic (e.g., compliance with rules) has shown that predictors have not been constant but rather context specific [15]. This effect may also be salient for psychological experiences and has important consequences for pandemic planning.

Therefore, drawing data from a large panel study of over 57,000 adults with a follow-up of 25 months, this study first aimed to show how depressive and anxiety symptoms changed by month between March 2020 and April 2022 in England. Second, it aimed to examine how changes in depressive and anxiety symptoms were associated with changes in time-varying contextual and individual-level factors. Moreover, we were interested to assess if any of these longitudinal associations differed across different stages of the pandemic. For this, fixed-effects (FE) models were fitted across 3 distinct periods between March 2020 and November 2021.

## Methods

### Data source and participants

This study analysed data from the University College London (UCL) COVID-19 Social Study (CSS), a large panel study of the psychological and social experiences of over 75,000 adults (aged 18+) in the United Kingdom (UK) during the COVID-19 pandemic. The study commenced on 21 March 2020 and involved weekly online data collection until August 2020 and then monthly (4-weekly) until November 2021. When the survey was converted to monthly, participants were randomly assigned into 4 groups. Each received the survey invitation and reminder in a different week of the month to have survey responses spread out over different days (averaging 970 participants per day during monthly data collection compared to 4,300 per day during weekly data collection). Following the monthly data collection, additional follow-ups were carried out in January and March 2022. The study did not use a random sample design, and therefore, the original sample is not representative of the UK adult population. However, the study had a 3-fold recruitment strategy that was designed to maximise the heterogeneity of the sample and to ensure representation from vulnerable and marginalised groups. First, convenience sampling was used, including promoting the study through existing networks and mailing lists (including large databases of adults who had previously consented to be involved in health research across the UK), print and digital media coverage, and social media. Second, more targeted recruitment was undertaken focusing on (i) individuals from a low-income background; (ii) individuals with no or few educational qualifications; and (iii) individuals who were unemployed. Third, the study was promoted via partnerships with third sector organisations to vulnerable groups, including adults with preexisting mental health conditions, older adults, carers, and people experiencing domestic violence or abuse. The study was approved by the UCL Research Ethics Committee [12467/005] and all participants gave written informed consent. A full protocol for the study is available via the UK Data Service.

This study focused on participants living in England (*N* = 59,810). After excluding participants with missing data in depressive or anxiety symptoms or any of variables included in weighting (see Statistical analysis below), we had an analytical sample of 57,766 unique participants and 950,113 total observations that were used in descriptive analyses, and smaller sample sizes in the main analysis (see S1 Fig).

### Measures

#### Mental health outcomes

Depressive symptoms were measured using the Patient Health Questionnaire (PHQ-9) [16], a standardised instrument for screening for depression in primary care. Unlike the original PHQ-9, the current study enquired about symptoms “over the last week” instead of “over the last 2 weeks” as data were initially collected weekly. The questionnaire includes 9 items with 4-point responses ranging from “not at all” to “nearly every day.” Higher overall scores indicate more depressive symptoms, ranging from 0 to 27.

Anxiety symptoms were measured using the Generalized Anxiety Disorder assessment (GAD-7) [17], a well-validated tool used to screen for generalised anxiety disorder in clinical practice and research. These questions were also worded as “over the last week” for the same reason as the PHQ-9 items. The GAD-7 comprises 7 items with 4-point responses ranging from “not at all” to “nearly every day,” with higher overall scores indicating more symptoms of anxiety, ranging from 0 to 21.

#### Contextual predictors

**Policy responses**. Stringency index was obtained from the Oxford COVID-19 Government Response Tracker (OxCGRT) [18]. It records the strictness of governmental policies that primarily restrict individual’s behaviours through a composite measure of 9 response metrics, such as stay-at-home orders, workplace closure, cancellation of public events, public information campaigns, and so forth. It ranges from 0 to 100, with a higher value indicating a stricter response. The index varies over time across the 4 devolved countries in the UK. This study used data exclusively from England. Vaccination was defined as the cumulative number of vaccines given including all doses by published date (in England) obtained from the UK government website for data on COVID-19 [19].

**Pandemic intensity**. Daily COVID-19 cases were defined as the number of new cases per day based on data published date. Daily COVID-19 deaths were defined as new deaths within 28 days of a positive test. Both measures (in England) were obtained from the UK government website [19]. All contextual variables were available on a daily basis that were linked to the individual data from CSS based on survey dates.

#### Individual predictors

At an individual level, we measured people’s confidence in the government, healthcare services, and access to essentials (e.g., access to food, water, medicines, deliveries). Participants were asked to rate their confidence levels for each on a scale of 1 (not at all confident) to 7 (very confident). Participants were also asked to rate their knowledge level of COVID-19 on a scale of 1 to 7. For COVID-19 stress, people were asked if they had been worrying about catching COVID-19 (0 = no, 1 = yes, minor stress, 2 = yes, major stress) or becoming seriously ill from COVID-19 (0 = no, 1 = yes, minor stress, 2 = yes, major stress). These were combined into a total stress score ranging from 0 to 4. Moreover, we included self-reported COVID-19 infection. During the weekly data collection (March to August 2020), participants were asked if they had had COVID-19, whereas during the 4-weekly phrase, they were asked if they had had COVID-19 in the past month. This was coded as a binary variable (0 = no/not know of, 1 = diagnosed/not formally diagnosed but suspected). Finally, we considered perceived social support as a mental health predictor. This was measured by an adapted version of the 6-item short form of Perceived Social Support Questionnaire, with a high reliability (α = 0.90) [20]. Each item was rated on a 5-point scale from “not true at all” to “very true.” A total score was generated based on confirmatory factor analysis, with higher scores indicating higher levels of support. All predictors (except for COVID-19 infection) were standardised to have a mean of 0 and standard deviation of 1 in the main analysis sample (*N* = 57,692, see S1 Fig). Full questions and responses for each item described above are available via the UK Data Service.

### Statistical analysis

We started with descriptive analyses showing mental health trends over time. Data were analysed in 4-week periods from March 2020 to November 2021, with additional follow-ups in January and March 2022. Weekly data between March to August 2020 were aggregated by taking means within each individual, and each wave of data were weighted separately to the proportions of gender, age, ethnicity, and education in the English adult population [21].

Next, data were analysed using FE models. FE analysis uses only within-individual variation, which automatically controls for observed or unobserved individual heterogeneities [22]. It addresses how the change in a predictor is related to the change in mental health outcomes. Further, we were interested in if any longitudinal association differed across time. Therefore, FE models were fitted separately across 3 periods based on study design and changes in policy responses (see S2 Fig): (i) first national lockdown (until the end of weekly data collection): 21/03/2020–23/08/2020 (weekly data, *N* = 45,838, mean number of time points (T_mean_) = 11.5, median number of time points (T_median_) = 12, see S1 Table); (ii) second and third national lockdown: 21/09/2020–11/04/2021 (4-weekly data, *N* = 26,175, T_mean_ = 6.1, T_median_ = 7, see S2 Table); (iii) “freedom” period: 12/04/2021–14/11/2021 (4-weekly data, *N* = 21,194, T_mean_ = 6.3, T_median_ = 7, see S3 Table). In each dataset for the FE analyses, we had further excluded participants with missing values in any of the predictors and those with less than 2 time points (see S1 Fig). These 3 datasets were weighted separately. To address multiplicity, we provided adjusted *p* values (q values) controlling for the positive false discovery rate, using the Benjamini–Yekutieli method implemented with the “qqvalue” command [23]. This took into account the total number of parameters across 3 periods for each outcome variable. In addition to the main analyses, we fitted the FE models using standardised outcomes. We also conducted sensitivity analyses testing the seasonal impact by including a winter dummy variable. All analyses were conducted using Stata V17. This study is reported as per the Strengthening the Reporting of Observational Studies in Epidemiology (STROBE) guideline.

## Results

### Descriptive statistics

Baseline characteristics of participants in different study periods are shown in S4 Table. After weighting, the 3 cohorts showed sociodemographic profiles in line with national statistics, for example, approximately 50% being women, 14% from ethnic minority backgrounds [21]. These and other time-invariant measures (observed or unobserved) were controlled for but not estimated in FE analyses. For time-varying measures, the within and between person variations are reported in Table 1. The mean depressive symptoms were slightly higher during the 2 lockdown periods (${\bar{\text{x}}}_{1}=5.76$, ${\bar{\text{x}}}_{2}=5.84$) compared to the “freedom” period (${\bar{\text{x}}}_{3}=5.69$). In all periods, the variation in depressive symptoms was mostly related to the differences between participants ($\hat{\rho }=0.82$). Similarly, anxiety symptoms also appeared to be higher during lockdowns, with variation mostly due to between person differences ($\hat{\rho }=0.81-0.82$). As expected, means of the contextual predictors varied substantially across 3 study periods. For example, national policies were the strictest during the second and third lockdowns period (${\bar{\text{x}}}_{2}=0.71$) and least restricted in the “freedom” period (${\bar{\text{x}}}_{3}=-1.13$). Daily COVID-19 cases were low during the first lockdown (${\bar{\text{x}}}_{1}=-0.40$), but much higher later on (${\bar{\text{x}}}_{2}=0.06$, ${\bar{\text{x}}}_{3}=0.12$). Daily deaths due to COVID-19 were higher during lockdown periods (${\bar{\text{x}}}_{1}=0.17$, ${\bar{\text{x}}}_{2}=0.41$), but much lower in the last study period (${\bar{\text{x}}}_{3}=-0.61$). This could be partially related to vaccination, which started on 8 December 2020. Therefore, vaccination was invariant in Period I and much lower in Period II than Period III. The variation in the contextual predictors was mostly related to differences within participants over time. For individual-level predictors, changes over time were also observed. However, most of variation in these variables was between rather than within participants.

**Table 1:** Descriptive statistics of time-varying variables across 3 periods (weighted).

	Period I: First lockdown (21/03/2020–23/08/2020) (*N*^†^ = 45,838, T^‡^ = 11.5)				Period II: Second and third lockdowns (21/09/2020–11/04/2021) (*N*^†^ = 26 175, T^‡^ = 6.1)				Period III: Freedom (12/04/2021–14/11/2021) (*N*^†^ = 21,194, T^‡^ = 6.3)			
	Mean ${\bar{\text{x}}}_{1}$	Between ${\hat{\sigma }}_{u1}$	Within ${\hat{\sigma }}_{e1}$	Ratio ${\hat{\rho }}_{1}$	Mean ${\bar{\text{x}}}_{2}$	Between ${\hat{\sigma }}_{u2}$	Within ${\hat{\sigma }}_{e2}$	Ratio ${\hat{\rho }}_{1}$	Mean ${\bar{\text{x}}}_{3}$	Between ${\hat{\sigma }}_{u3}$	Within ${\hat{\sigma }}_{e3}$	Ratio ${\hat{\rho }}_{1}$
Depressive symptoms	5.76	5.36	2.50	0.82	5.84	5.18	2.44	0.82	5.69	5.06	2.37	0.82
Anxiety symptoms	4.29	4.77	2.22	0.82	4.40	4.54	2.18	0.81	4.10	4.38	2.10	0.81
Stringency index (std)	0.35	0.32	0.44	0.34	0.71	0.15	0.51	0.08	-1.13	0.41	1.00	0.14
Vaccination (std)	-0.55	0.00	0.00	--	-0.34	0.12	0.31	0.12	1.30	0.19	0.45	0.16
New cases per day (std)	-0.40	0.03	0.03	0.39	0.06	0.11	0.42	0.07	0.12	0.17	0.42	0.14
New deaths per day (std)	0.17	0.69	0.96	0.34	0.41	0.45	1.28	0.11	-0.61	0.07	0.18	0.14
Confidence: government (std)	0.18	0.87	0.48	0.77	-0.06	0.91	0.41	0.83	0.09	0.96	0.38	0.87
Confidence: healthcare (std)	0.12	0.85	0.55	0.70	-0.02	0.87	0.57	0.70	0.14	0.88	0.51	0.75
Confidence: essential (std)	0.06	0.84	0.58	0.68	0.10	0.85	0.54	0.71	-0.00	0.86	0.70	0.60
COVID-19 knowledge (std)	-0.07	0.91	0.52	0.76	-0.07	0.90	0.46	0.79	-0.04	0.90	0.43	0.81
COVID-19 stress (std)	0.08	0.92	0.59	0.71	0.01	0.86	0.55	0.71	-0.23	0.72	0.46	0.71
COVID-19 infection	0.11	0.32	0.13	0.86	0.04	0.13	0.16	0.40	0.03	0.10	0.13	0.37
Social support (std)	-0.10	0.87	0.37	0.85	-0.03	0.92	0.32	0.89	-0.02	0.94	0.31	0.90

All predictors were standardised (std) in the main analysis sample (before splitting into different periods) to have a mean of 0 and standard deviation of 1, except for the binary variable, COVID-19 infection.

^†^ Number of unique participants.

^‡^ Number of time points (week/month) per participant.

### Mental health changes over time

**Fig 1:**
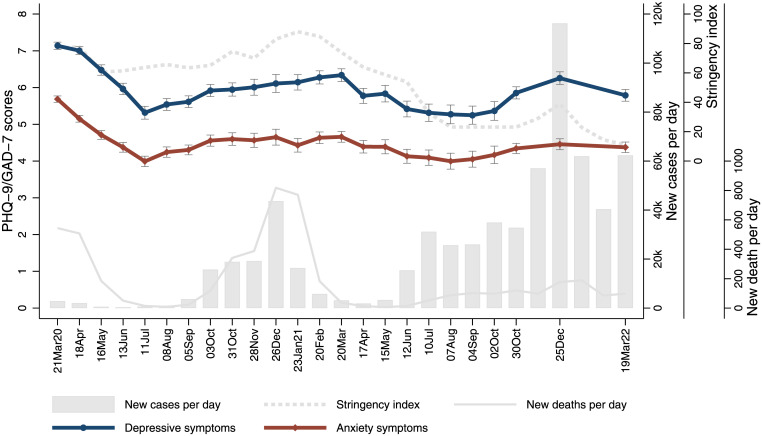
Trends of depressive and anxiety symptoms (weighted means) in relation to policy responses (stringency index) and pandemic intensity (daily COVID-19 cases and deaths) between March 2020 and April 2022. COVID-19, Coronavirus Disease 2019; GAD, generalized anxiety disorder.

Fig 1 shows the weighted means of anxiety and depressive symptoms and their 95% confidence interval (CI) from each wave in relation to stringency index, average daily COVID-19 cases, and deaths (see S3 Fig for categorised measures). Both depressive and anxiety symptoms were high at the start of the first national lockdown, but decreased rapidly in the first few months before starting to raise again in August 2020 until the end of the third national lockdown in March 2021. Both measures then decreased gradually into the summer months of 2021, but then showed increases towards December 2021-January 2022. This is particularly the case for depressive symptoms which lowered again in March to April 2022. These trends were consistent with descriptive statistics presented above, but showing detailed gradients in changes over time.

### Longitudinal predictors of changes in mental health

**Fig 2:**
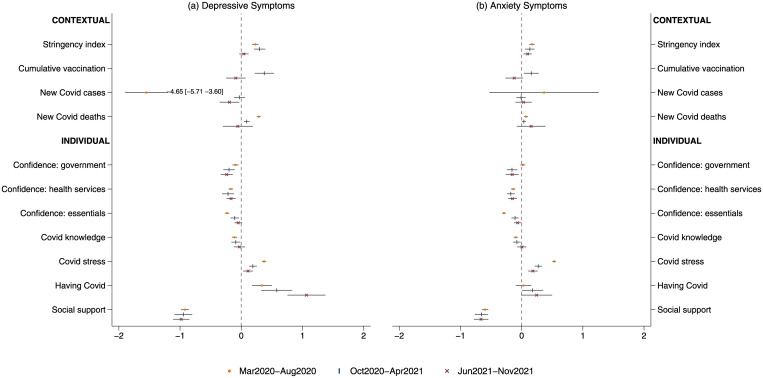
Estimated coefficients and 95% CIs from FE models of 3 time periods during the COVID-19 pandemic. Notes: The estimates for new COVID case from models on depressive symptoms were on a different scale compared to other predictors in order to fit into the figure without changing the x-axis scale. CI, confidence interval; COVID-19, Coronavirus Disease 2019; FE, fixed-effects.

As expected, most contextual measures were closely related to each other (S4 Fig; see S5 Table for multicollinearity diagnostics). Therefore, it is important to examine the longitudinal association between each predictor and the outcomes while controlling for other factors. The results from FE models including both contextual and individual-level factors are reported in Table 2 and Fig 2 (see S6 Table for FE models with contextual predictors only). Both *p* and q values are reported in Table 2. We would refer to q values when interpreting the results.

**Table 2:** Results from FE models across 3 periods (weighted).

	Period I: First lockdown (21/03/2020–23/08/2020) (*N*^†^ = 45 838, T^‡^_mean_ = 11.5)					Period II: Second and third lockdowns (21/09/2020–11/04/2021) (*N*^†^ = 26,175, T^‡^_mean_ = 6.1)					Period III: Freedom (12/04/2021–14/11/2021) (*N*^†^ = 21,194, T^‡^_mean_ = 6.3)				
	Coef.	95% CI		p	q	Coef.	95% CI		p	q	Coef.	95% CI		p	q
**Depressive symptoms**															
Stringency index (std)	0.23	0.18	0.28	<0.001	<0.001	0.30	0.21	0.39	<0.001	<0.001	0.04	-0.03	0.12	0.262	1.000
Vaccination (std)	--	--	--	--	--	0.38	0.22	0.53	<0.001	<0.001	-0.09	-0.25	0.07	0.271	1.000
New cases per day (std)	-4.65	-5.71	-3.60	<0.001	<0.001	-0.03	-0.12	0.06	0.560	1.000	-0.19	-0.35	-0.03	0.018	0.091
New deaths per day (std)	0.29	0.25	0.32	<0.001	<0.001	0.09	0.05	0.13	<0.001	0.001	-0.06	-0.30	0.19	0.655	1.000
Confidence: government (std)	-0.09	-0.14	-0.04	<0.001	0.001	-0.20	-0.29	-0.11	<0.001	<0.001	-0.24	-0.34	-0.14	<0.001	<0.001
Confidence: healthcare (std)	-0.17	-0.21	-0.13	<0.001	<0.001	-0.22	-0.31	-0.12	<0.001	<0.001	-0.17	-0.24	-0.09	<0.001	<0.001
Confidence: essential (std)	-0.23	-0.27	-0.19	<0.001	<0.001	-0.11	-0.18	-0.04	0.002	0.012	-0.05	-0.11	0.02	0.139	0.668
COVID-19 knowledge (std)	-0.11	-0.15	-0.07	<0.001	<0.001	-0.09	-0.16	-0.02	0.017	0.086	-0.03	-0.12	0.06	0.501	1.000
COVID-19 stress (std)	0.37	0.34	0.41	<0.001	<0.001	0.19	0.13	0.25	<0.001	<0.001	0.11	0.03	0.19	0.007	0.038
COVID-19 infection	0.34	0.18	0.50	<0.001	<0.001	0.58	0.33	0.83	<0.001	<0.001	1.07	0.75	1.38	<0.001	<0.001
Social support (std)	-0.92	-0.98	-0.86	<0.001	<0.001	-0.95	-1.09	-0.80	<0.001	<0.001	-0.98	-1.11	-0.85	<0.001	<0.001
**Anxiety symptoms**															
Stringency index (std)	0.17	0.12	0.21	<0.001	<0.001	0.13	0.06	0.21	0.001	0.005	0.10	0.03	0.17	0.005	0.035
Vaccination (std)	--	--	--	--	--	0.16	0.04	0.28	0.011	0.078	-0.12	-0.26	0.02	0.100	0.519
New cases per day (std)	0.37	-0.53	1.26	0.422	1.000	-0.01	-0.09	0.07	0.799	1.000	0.03	-0.10	0.16	0.644	1.000
New deaths per day (std)	0.07	0.04	0.10	<0.001	<0.001	0.04	0.00	0.07	0.030	0.184	0.16	-0.08	0.39	0.192	0.961
Confidence: government (std)	0.02	-0.02	0.06	0.359	1.000	-0.16	-0.24	-0.07	<0.001	0.002	-0.15	-0.26	-0.05	0.004	0.032
Confidence: healthcare (std)	-0.14	-0.17	-0.10	<0.001	<0.001	-0.18	-0.24	-0.11	<0.001	<0.001	-0.15	-0.22	-0.08	<0.001	<0.001
Confidence: essential (std)	-0.29	-0.32	-0.26	<0.001	<0.001	-0.11	-0.17	-0.05	<0.001	0.003	-0.06	-0.13	0.00	0.035	0.209
COVID-19 knowledge (std)	-0.10	-0.13	-0.06	<0.001	<0.001	-0.08	-0.14	-0.02	0.013	0.087	0.00	-0.07	0.08	0.909	1.000
COVID-19 stress (std)	0.53	0.50	0.56	<0.001	<0.001	0.27	0.21	0.33	<0.001	<0.001	0.18	0.11	0.26	<0.001	<0.001
COVID-19 infection	0.03	-0.10	0.16	0.645	1.000	0.18	0.00	0.35	0.044	0.247	0.24	-0.01	0.50	0.058	0.315
Social support (std)	-0.60	-0.65	-0.55	<0.001	<0.001	-0.66	-0.76	-0.55	<0.001	<0.001	-0.66	-0.78	-0.54	<0.001	<0.001

All predictors were standardised (std) in the total sample to have a mean of 0 and standard deviation of 1, except for the binary variable, COVID-19 infection.

^†^ Number of unique participants.

^‡^ Mean number of time points (week/month) per participant.

CI, confidence interval; COVID-19, Coronavirus Disease 2019; FE, fixed-effects; std, standardised.

#### Depressive symptoms

During Periods I and II, 1 standardised deviation (SD) increase in stringency index was associated with 0.23 to 0.30 points increase in depressive symptoms. But there was no evidence for this longitudinal association in Period III. An increase in COVID-19 cases was associated with a decrease in depressive symptoms, but only in Period I. An increase in COVID-19 deaths was associated with higher depressive symptoms, but this abated over time. One SD increase in vaccination was associated with a 0.38-point increase in depressive symptoms in Period II, without any evidence in Period III.

There was a negative longitudinal association between confidence in government and depressive symptoms across all study periods, with the magnitude of the association increasing over time. A negative longitudinal association with depressive symptoms was also found for confidence in healthcare services in all periods and for confidence in access to essentials in Periods I and II (although the latter decreased in strength over time). One SD increase in COVID-19 knowledge was associated with a 0.11-point decrease in depressive symptoms in Period I. But no evidence was found in Periods II and III. An increase in COVID-19-related stress was associated with an increase in depressive symptoms, with the association being smaller in later periods. COVID-19 infection was associated with increases in depressive symptoms in all periods, with this association becoming stronger in later periods. Finally, increases in social support were consistently associated with decreases in depressive symptoms.

#### Anxiety symptoms

Increases in stringency index were associated with increases in anxiety symptoms in all study periods. There was no evidence for a longitudinal association of anxiety with COVID-19 cases. However, 1 SD increase in COVID-19 deaths was associated a 0.07-point increase in anxiety, but in Period I only. There was limited evidence for a longitudinal association between national vaccination and anxiety. There was no evidence that confidence in government was associated with anxiety in Period I, but significant longitudinal associations were found in Periods II and III. A negative longitudinal association of anxiety with confidence in healthcare services was found in all study periods, whereas its relationship with confidence in essentials was significant in Periods I and II only. Increased COVID-19 knowledge was associated with lower anxiety in Period I, with limited evidence in Periods II and III. Increases in COVID-19 stress were associated with higher anxiety symptoms consistently, although the association became less prominent in later periods. There was no evidence for a longitudinal association between COVID-19 infection and anxiety. Finally, increased social support was associated with lower anxiety symptoms across time.

S7 Table reports the results from FE models with standardised outcomes. The standardised longitudinal coefficients were mostly small, except for COVID-19 cases in period I (β_depressive_ = −0.83), COVID-19 infection in period II (β_depressive_ = 0.10), and III (β_depressive_ = 0.19), and perceived social support (β_depressive_ = −0.16-(−0.18), β_anxiety_ = −0.12-(−0.13). S8 Table shows sensitivity analysis for Period II accounting for seasonality by including a winter dummy (November 2020 to March 2021). The results were largely consistent.

## Discussion

Drawing data from a large panel study with regular follow-ups, this study showed how depressive and anxiety symptoms changed by month over 2 years between March 2020 and April 2022 in England. To our knowledge, this makes it the study with the longest and most frequent mental health follow-ups after the outbreak of the COVID-19 pandemic in England. Both depressive and anxiety symptoms were high at the start of the first national lockdown, but fell rapidly in the first few months. However, they rose in late 2020 to early 2021 as the number of cases increased and COVID-19 restrictions tightened before deceasing again following the end of the third national lockdown, despite relatively high numbers of COVID-19 cases. This is generally in line with findings from other data [14]. In addition, our study showed that there was a rise in depressive symptoms, in particular, in late 2021 when case numbers peaked, even though no further lockdowns were brought in. Further, our analyses showed how changes in contextual and individual level factors were associated with changes in depressive and anxiety symptoms across different stages of the pandemic, including the first national lockdown, the second and third national lockdowns, and the period of minimal restrictions.

Several factors were related to both depressive and anxiety symptoms consistently across all study periods. These included confidence in healthcare services, COVID-19 related stress, and perceived social support. In the face of the COVID-19 crisis, the National Health Service (NHS) has been under extreme pressure because of surging demands and staff absences [24]. This was reinforced by the fact that “protect the NHS” was communicated as part of the key public messages at the COVID-19 press briefings at the early stage of the pandemic. Therefore, it is not surprising that people’s confidence in how the NHS coped during the pandemic was related to depressive and anxiety symptoms, regardless of time periods. Additionally, there was evidence to suggest that during the pandemic, many people experienced disruptions to accessing healthcare services or reductions in help-seeking due to fear of COVID-19 infection or feeling that they should not burden the health service [25,26]. So perceived unavailability of mental health support due to overall load on the health service could also explain the relationship with higher anxiety and depressive symptoms. It is notable that the relationship between stress about COVID-19 and mental health symptoms weakened over time (even though it remained significant and, compared to other standardised predictors, meaningful in size). This was likely influenced by the roll-out of vaccinations, which reduced the risk of hospitalisation and deaths from the virus. However, the associations with stress about COVID-19 were independent of the vaccination variable. Therefore, the diminishing relationship between COVID-19 stress and mental health over time is likely at least in part due to habituation, whereby the ongoing exposure to information on COVID-19 and increased number of contacts who had contracted and survived COVID-19 reduced the psychological salience of the stressor [27]. Our findings on perceived social support are in line with pre-pandemic literature showing the mental health benefits of social relationships [28,29]. They suggest that social support continued to play an important role in supporting people’s mental health during the heightened emotional distress and psychological challenges of the COVID-19 pandemic. In fact, social support was arguably the most important predictor overall when comparing coefficients across standardised predictors.

Other factors showed differential longitudinal association with depressive and/or anxiety symptoms across different stages of the pandemic. For example, increases in COVID-19 policy measures were associated with increased depressive symptoms during lockdown periods, but no evidence was found after the easing of restrictions following the third national lockdown. This could be because policy changes at this stage were relatively minor, which had limited impact on personal freedom especially after most legal restrictions being removed in July 2021. It also suggests that the relationship between policy stringency and mental health may not be proportionate, with increases in policy changes becoming more damaging for mental health as they infringe more on social freedoms. Increased COVID-19 deaths were associated with higher depressive and anxiety symptoms only at early stages of the pandemic. This might be explained by the relatively low and stable death rate in the “freedom” period possibly due to the roll out of vaccination programme, so that deaths from COVID-19 no longer presented itself as a major psychological threat. However, after controlling for other contextual factors, our analyses showed that progresses in vaccination programme was associated with higher depressive symptoms and anxiety (nonsignificant q value) in the second study period. The vaccination programme in England began on 8 December 2020. There had been significant vaccine scepticism and hesitancy at the start [24,30], which may explain the positive longitudinal association. However, with effective communication of scientific information on COVID-19 vaccination over time, this association was no longer observed at the later stage.

For individual-level factors, increased confidence in accessing essentials was associated with reduced depressive and anxiety symptoms only during lockdown periods, particularly the first national lockdown when essential and non-essential services were most heavily influenced either due to COVID-19 restrictions or staff shortage. Finally, an increase in self-rated COVID-19 knowledge was associated with decreased depressive and anxiety symptoms, but only in the first lockdown period. A plausible explanation is that knowledge about the virus and its transmission was particularly salient at the start when there was a general lack of understanding accompanied by excessive circulation of misinformation [31].

Although depressive and anxiety symptoms are highly correlated with each other, it is important to acknowledge differences in how they are related to some of the predictors. Our findings showed that COVID-19 infection was associated with increased depressive symptoms consistently across all study periods, but limited evidence was found for anxiety. Notably, this association was independent of stress about COVID-19. Further, it is interesting that the association was for having COVID-19 oneself rather than the overall national case rates of COVID-19. This suggests that individual psychobiological mechanisms of COVID-19 could be responsible for the association with depressive symptoms. Existing literature proposes that inflammatory mechanisms in COVID-19 infection could be responsible for an increase in depressive symptoms [32,33]. This could certainly resonate with the association only being found for depression, and not for anxiety [34]. Although there was no evidence for the longitudinal association of stringency index with depressive symptoms in the “freedom” period, the association was found for anxiety symptoms. It is possible that easing of restrictions at this stage despite being inconsequential for depressive symptoms, still had some impact on anxiety, relating to COVID-19 or other challenges. Moreover, confidence in government was associated with depressive symptoms in all periods, but no evidence was found during the first national lockdown period for anxiety. Relatively speaking, there was a higher level of support for COVID-19 policy responses at the start of the pandemic as shown in confidence in government [35], whereas people disagreed to a greater extent with the government at later stages (Table 1). Therefore, it is possible that people were less anxious in relation to confidence in the government (after controlling for policy stringency) during the first national lockdown when confidence in their capability was higher.

This study has a number of strengths. It utilised a large sample with sufficient heterogeneity to include good stratification across all major sociodemographic groups and good coverage of geographic areas in England (S1 Table). The analyses were weighted on the basis of population estimates of core demographics, with the weighted data showing good alignment with national population estimates. Due to the longitudinal design of the COVID-19 Social Study, we were able to examine overall trends of depressive and anxiety symptoms over 25 months and longitudinal predictors of changes in them across different stages of the COVID-19 pandemic. However, our study is not without any limitation. First, it is important to acknowledge that our data were from a non-probability sample. Despite the effort to recruit a heterogeneous sample and make our sample representative to the adult population in England by weighting, there is still the possibility of potential biases due to omitting other demographic factors that could be associated with survey participation in the weighting process. One example is the difference in internet access that is not explained by variables such as age and education. It is possible that these findings might not be generalisable to the population. Relatedly, although the 3 periods had very similar sample characteristics after weighting, we could not account for unmeasured confounders which might influence the comparability across periods. Second, the FE approach cannot rule out the possibility of bidirectional associations of depressive/anxiety symptoms with individual-level factors. This calls for further research in this area. Third, due to a lack of pre-pandemic information, it is unclear in our study per se how mental health trends during COVID-19 are related to patterns before the pandemic. Moreover, future research is also needed to extend the follow-up period, showing mental health changes beyond the COVID-19 pandemic. Finally, general assessment tools such as PHQ-9 and GAD-7 might not capture psychological distress related to COVID-19 specifically nor wider psychiatric disorders [36]. Therefore, future research using broader instruments including those and taking into account the COVID-19 context is recommended.

Since July 2021, England removed most of its legal restrictions, making it one of the countries with the least strict COVID-19 policies and highest vaccine coverage at the time of writing [37]. The United Kingdom is also one of the countries with highest cumulative number of COVID-19 cases and deaths [37]. But there is a lot of variation in COVID-19 situation and policy stringency across countries and over time. Our results provided empirical evidence on how changes in contextual measures, including stringency index, COVID-19 cases, COVID-19 deaths, and national vaccination, as well as individual level factors, such as COVID-19 related stress, COVID-19 infection, and social support were related to depressive and anxiety symptoms. While some factors (especially individual factors) clearly act as consistent predictors of mental health during a pandemic (e.g., social support, stress about catching the virus, and confidence in government, health services and access to essentials), other factors (especially contextual factors) are dependent on the specific situations occurring within society as to whether they impact mental health or not (e.g., policy stringency, cases, deaths, and vaccination rates). This could provide important implications for policy making and for a better understanding of mental health of the general public during a national or global health crisis.

## Supporting information

S1 File STROBE Checklist. (DOCX)

S1 Table Number of observations and follow-up rates by week (period I). (DOCX)

S2 Table Number of observations and follow-up rates by month (period II). (DOCX)

S3 Table Number of observations and follow-up rates by month (period III). (DOCX)

S4 Table Baseline sample characteristics across 3 study periods. (DOCX)

S5 Table Multicollinearity diagnostics across study periods (unweighted). (DOCX)

S6 Table Results from fixed-effects models with contextual factors only across 3 periods (weighted). (DOCX)

S7 Table Results from fixed-effects models with standardised outcomes across 3 periods (weighted). (DOCX)

S8 Table Results from fixed-effects models comparing Period II models with and without seasonal dummy (weighted). (DOCX)

S1 Fig Sample selection diagram (*N* = number of unique participants, T = number of time points). (DOCX)

S2 Fig Daily changes in COVID-19 policy responses (stringency index) in England from January 2020 to April 2022. (DOCX)

S3 Fig Trends of depressive (weighted percentages in 5 categories) and anxiety symptoms (weighted percentages in 4 categories) over time from March 2020 to March 2022. (DOCX)

S4 Fig Correlation matrix of predictors across study periods (unweighted). (DOCX)

## Abbreviations

CI: confidence interval
COVID-19: Coronavirus Disease 2019
CSS: COVID-19 Social Study
FE: fixed-effects
GAD: generalized anxiety disorder
NHS: National Health Service
UCL: University College London
