# Supplementary material for: Depressive and anxiety symptoms in adults during the COVID-19 pandemic in England: A panel data analysis over 2 years
Source: PLoS Med. 2023 Apr 18;20(4):e1004144. doi: 10.1371/journal.pmed.1004144 (PMC10112796; doi:10.1371/journal.pmed.1004144)
Supplement: S1 Table — (DOCX) [file pmed.1004144.s002.docx]

S1 Table Number of observations and follow-up rates by week (period I)

| Week | Date | Frequency | % of total Obs. | % with follow-ups | |
| --- | --- | --- | --- | --- | --- |
| 1 | 21/03/2020-27/03/2020 | 19,386 | 3.68 | 100.00 |  |
| 2 | 28/03/2020-03/04/2020 | 22,707 | 4.31 | 93.60 |  |
| 3 | 04/04/2020-10/04/2020 | 29,286 | 5.56 | 94.67 |  |
| 4 | 11/04/2020-17/04/2020 | 29,450 | 5.59 | 92.89 |  |
| 5 | 18/04/2020-24/04/2020 | 28,837 | 5.47 | 90.01 |  |
| 6 | 25/04/2020-01/05/2020 | 27,359 | 5.19 | 91.35 |  |
| 7 | 02/05/2020-08/05/2020 | 27,776 | 5.27 | 94.70 |  |
| 8 | 09/05/2020-15/05/2020 | 28,885 | 5.48 | 92.79 |  |
| 9 | 16/05/2020-22/05/2020 | 27,748 | 5.27 | 94.47 |  |
| 10 | 23/05/2020-29/05/2020 | 26,187 | 4.97 | 95.13 |  |
| 11 | 30/05/2020-05/06/2020 | 25,389 | 4.82 | 95.75 |  |
| 12 | 06/06/2020-12/06/2020 | 24,727 | 4.69 | 95.94 |  |
| 13 | 13/06/2020-19/06/2020 | 23,893 | 4.54 | 96.20 |  |
| 14 | 20/06/2020-26/06/2020 | 23,000 | 4.37 | 96.57 |  |
| 15 | 27/06/2020-03/07/2020 | 22,540 | 4.28 | 96.29 |  |
| 16 | 04/07/2020-10/07/2020 | 21,694 | 4.12 | 96.42 |  |
| 17 | 11/07/2020-17/07/2020 | 21,005 | 3.99 | 96.41 |  |
| 18 | 18/07/2020-24/07/2020 | 20,443 | 3.88 | 96.39 |  |
| 19 | 25/07/2020-31/07/2020 | 19,648 | 3.73 | 96.39 |  |
| 20 | 01/08/2020-07/08/2020 | 19,255 | 3.65 | 96.30 |  |
| 21 | 08/08/2020-14/08/2020 | 18,689 | 3.55 | 89.92 |  |
| 22 | 15/08/2020-21/08/2020 | 18,073 | 3.43 | 4.72^‡^ |  |
| 23 | 22/08/2020-23/08/2020^†^ | 853^†^ | 0.16^†^ | -- |  |

Notes: † Incomplete week (only 2 days) due to date constraints for study periods, ^‡^ low rate due to truncated follow-up in week 23
