# Supplementary material for: Depressive and anxiety symptoms in adults during the COVID-19 pandemic in England: A panel data analysis over 2 years
Source: PLoS Med. 2023 Apr 18;20(4):e1004144. doi: 10.1371/journal.pmed.1004144 (PMC10112796; doi:10.1371/journal.pmed.1004144)
Supplement: S2 Table — (DOCX) [file pmed.1004144.s003.docx]

S2 Table Number of observations and follow-up rates by month (period II)

| Month | Date | Frequency | % of total Obs. | % with follow-ups | |
| --- | --- | --- | --- | --- | --- |
| 1 | 21/09/2020-02/10/2020^†^ | 12,312^†^ | 7.68^†^ | 100.00^†^ |  |
| 2 | 03/10/2020-30/10/2020 | 24,107 | 15.04 | 98.55 |  |
| 3 | 31/10/2020-27/11/2020 | 22,982 | 14.34 | 96.72 |  |
| 4 | 28/11/2020-25/12/2020 | 20,902 | 13.04 | 98.12 |  |
| 5 | 26/12/2020-22/01/2021 | 20,900 | 13.04 | 98.39 |  |
| 6 | 23/01/2021-19/02/2021 | 20,152 | 12.58 | 96.50 |  |
| 7 | 20/02/2021-19/03/2021 | 21,479 | 13.4 | 71.03^‡^ |  |
| 8 | 20/03/2021-11/04/2021^†^ | 17,416^†^ | 10.87^†^ | -- |  |

Notes: † Incomplete month due to date constraints for study periods, ^‡^ low rate due to truncated follow-up in month 8
