# Supplementary material for: Depressive and anxiety symptoms in adults during the COVID-19 pandemic in England: A panel data analysis over 2 years
Source: PLoS Med. 2023 Apr 18;20(4):e1004144. doi: 10.1371/journal.pmed.1004144 (PMC10112796; doi:10.1371/journal.pmed.1004144)
Supplement: S3 Table — (DOCX) [file pmed.1004144.s004.docx]

S3 Table Number of observations and follow-up rates by month (period III)

| Month | Date | Frequency | % of total Obs. | % with follow-ups | |
| --- | --- | --- | --- | --- | --- |
| 1 | 12/04/2021-16/04/2021^†^ | 4,310^†^ | 3.22^†^ | 100.00^†^ |  |
| 2 | 17/04/2021-14/05/2021 | 20,202 | 15.09 | 98.66 |  |
| 3 | 15/05/2021-11/06/2021 | 19,819 | 14.8 | 93.32 |  |
| 4 | 12/06/2021-09/07/2021 | 18,569 | 13.87 | 94.51 |  |
| 5 | 10/07/2021-06/08/2021 | 17,746 | 13.25 | 94.42 |  |
| 6 | 07/08/2021-03/09/2021 | 16,689 | 12.46 | 95.27 |  |
| 7 | 04/09/2021-01/10/2021 | 15,997 | 11.95 | 95.01 |  |
| 8 | 02/10/2021-29/10/2021 | 15,408 | 11.51 | 29.58^‡^ |  |
| 9 | 30/10/2021-15/11/2021^†^ | 5,152^†^ | 3.85^†^ | -- |  |

Notes: † Incomplete month due to date constraints for study periods ^‡^ low rate due to truncated follow-up in month 9
