# Supplementary material for: Depressive and anxiety symptoms in adults during the COVID-19 pandemic in England: A panel data analysis over 2 years
Source: PLoS Med. 2023 Apr 18;20(4):e1004144. doi: 10.1371/journal.pmed.1004144 (PMC10112796; doi:10.1371/journal.pmed.1004144)
Supplement: S4 Table — (DOCX) [file pmed.1004144.s005.docx]

S4 Table Baseline sample characteristics across three study periods

|  | Period I: 1^st^ lockdown  (21/03/2020-23/08/2020)  (N_1_=45,838) | | | Period II: 2^nd^ & 3^rd^ lockdowns  (21/09/2020-11/04/2021)  (N_2_=26,175) | | | Period III: freedom  (12/04/2021-14/11/2021)  (N_3_=21,194) | |
| --- | --- | --- | --- | --- | --- | --- | --- | --- |
|  | | unweighted | weighted | | unweighted | weighted | unweighted | weighted |
| **Gender** | |  |  | |  |  |  |  |
| Women | | 75.7% | 50.2% | | 75.5% | 50.5% | 75.0% | 50.1% |
| Men | | 24.3% | 49.8% | | 24.5% | 49.5% | 25.0% | 49.9% |
| **Age** | |  |  | |  |  |  |  |
| 18-29 | | 8.6% | 18.4% | | 4.8% | 17.6% | 4.0% | 17.5% |
| 30-45 | | 30.8% | 27.7% | | 22.6% | 27.7% | 20.5% | 27.9% |
| 46-59 | | 32.4% | 24.4% | | 33.8% | 24.7% | 33.6% | 24.7% |
| 60+ | | 28.1% | 29.6% | | 38.8% | 30.1% | 41.8% | 29.9% |
| **Ethnicity** | |  |  | |  |  |  |  |
| Ethnic minority groups | | 5.4% | 14.2% | | 4.2% | 14.0% | 3.9% | 14.1% |
| White | | 94.6% | 85.8% | | 95.8% | 86.0% | 96.1% | 85.9% |
| **Education** | |  |  | |  |  |  |  |
| Low (GCSEs or below) | | 13.0% | 32.1% | | 13.4% | 31.9% | 13.9% | 31.8% |
| Medium (A-levels or equivalent) | | 17.1% | 32.6% | | 16.6% | 31.9% | 16.8% | 32.1% |
| High (Degree or above) | | 69.9% | 35.3% | | 70.0% | 36.1% | 69.3% | 36.1% |
| **Household income** | |  |  | |  |  |  |  |
| <30k | | 36.4% | 45.9% | | 39.1% | 46.2% | 40.5% | 46.9% |
| ≥30k | | 63.6% | 54.1% | | 60.9% | 53.8% | 59.5% | 53.1% |
| **Employed status** | |  |  | |  |  |  |  |
| Employed | | 67.4% | 60.2% | | 60.9% | 59.1% | 58.9% | 59.8% |
| Other | | 32.6% | 39.8% | | 39.1% | 40.9% | 41.1% | 40.2% |
| **Mental health diagnosis** | |  |  | |  |  |  |  |
| Yes | | 18.8% | 20.1% | | 16.1% | 19.2% | 15.6% | 19.0% |
| No | | 81.2% | 79.9% | | 83.9% | 80.8% | 84.4% | 81.0% |
| **Regions†** | |  |  | |  |  |  |  |
| North East England | | 4.2% | 4.3% | | 4.2% | 4.2% | 4.3% | 4.2% |
| North West England | | 9.8% | 9.8% | | 9.9% | 9.6% | 9.8% | 9.9% |
| Yorkshire and the Humber | | 8.0% | 7.7% | | 8.2% | 7.8% | 8.3% | 8.0% |
| West Midlands | | 8.0% | 8.5% | | 8.1% | 8.5% | 8.1% | 8.4% |
| East Midlands | | 7.1% | 7.8% | | 7.1% | 8.2% | 7.2% | 7.6% |
| East of England | | 9.8% | 10.7% | | 10.0% | 10.8% | 10.0% | 11.1% |
| London | | 19.7% | 18.9% | | 19.2% | 19.4% | 18.7% | 19.9% |
| South East England | | 20.9% | 20.4% | | 20.8% | 19.9% | 20.9% | 19.4% |
| South West England | | 12.6% | 11.9% | | 12.5% | 11.6% | 12.7% | 11.5% |

Notes: Information on region is available for a smaller sample of participants as it was collected at a later stage of the COVID-19 Social Study (N_1_=30,241, N_2_=23,874, N_3_=20,498).
