# Supplementary material for: Depressive and anxiety symptoms in adults during the COVID-19 pandemic in England: A panel data analysis over 2 years
Source: PLoS Med. 2023 Apr 18;20(4):e1004144. doi: 10.1371/journal.pmed.1004144 (PMC10112796; doi:10.1371/journal.pmed.1004144)
Supplement: S5 Table — (DOCX) [file pmed.1004144.s006.docx]

S5 Table Multicollinearity diagnostics across study periods (unweighted)

|  | Period I  (n=526,818) | | Period II  (n=160,250) | | Period III  (n=133,892) | |
| --- | --- | --- | --- | --- | --- | --- |
|  | VIF | Tolerance | VIF | Tolerance | VIF | Tolerance |
| Stringency index (std) | 2.95 | 0.34 | 2.77 | 0.36 | 6.11 | 0.16 |
| Vaccination (std) | -- | -- | 2.40 | 0.42 | 7.06 | 0.14 |
| New cases per day (std) | 5.86 | 0.17 | 2.03 | 0.49 | 4.07 | 0.25 |
| New deaths per day (std) | 4.16 | 0.24 | 2.62 | 0.38 | 2.13 | 0.47 |
| Confidence: government (std) | 1.41 | 0.71 | 1.28 | 0.78 | 1.30 | 0.77 |
| Confidence: healthcare (std) | 1.80 | 0.56 | 1.84 | 0.54 | 1.83 | 0.55 |
| Confidence: essential (std) | 1.69 | 0.59 | 1.81 | 0.55 | 2.02 | 0.50 |
| COVID knowledge (std) | 1.06 | 0.95 | 1.04 | 0.96 | 1.05 | 0.95 |
| COVID stress (std) | 1.07 | 0.93 | 1.08 | 0.93 | 1.06 | 0.94 |
| COVID infection | 1.01 | 0.99 | 1.00 | 1.00 | 1.00 | 1.00 |
| Social support (std) | 1.06 | 0.94 | 1.06 | 0.94 | 1.06 | 0.95 |

Notes: A large value of VIF indicates a higher level of linear dependency. The threshold value of 10 is generally taken as indicating multicollinearity.
