# Supplementary material for: Depressive and anxiety symptoms in adults during the COVID-19 pandemic in England: A panel data analysis over 2 years
Source: PLoS Med. 2023 Apr 18;20(4):e1004144. doi: 10.1371/journal.pmed.1004144 (PMC10112796; doi:10.1371/journal.pmed.1004144)
Supplement: S6 Table — (DOCX) [file pmed.1004144.s007.docx]

S6 Table Results from fixed effects models with contextual factors only across three periods (weighted)

|  | Period I: 1^st^ lockdown  (21/03/2020-23/08/2020)  (N^†^=45,838, T^‡^_mean_=11.5) | | | | | Period II: 2^nd^ & 3^rd^ lockdowns  (21/09/2020-11/04/2021)  (N^†^=26,175, T^‡^_mean_=6.1) | | | | | Period III: freedom  (12/04/2021-14/11/2021)  (N^†^=21,194, T^‡^_mean_=6.3) | | | | |
| --- | --- | --- | --- | --- | --- | --- | --- | --- | --- | --- | --- | --- | --- | --- | --- |
|  | Coef. | 95% CI | | p | q | Coef. | 95% CI | | p | q | Coef. | 95% CI | | p | q |
| **Depressive symptoms** |  |  |  |  |  |  |  |  |  |  |  |  |  |  |  |
| Stringency index (std) | 0.37 | 0.32 | 0.42 | <0.001 | <0.001 | 0.25 | 0.16 | 0.34 | <0.001 | <0.001 | 0.03 | -0.05 | 0.10 | 0.448 | 1.000 |
| Vaccination (std) | -- | -- | -- | -- | -- | 0.13 | -0.02 | 0.28 | 0.086 | 0.391 | -0.03 | -0.19 | 0.13 | 0.679 | 1.000 |
| New cases per day (std) | -4.45 | -5.53 | -3.37 | <0.001 | <0.001 | 0.11 | 0.03 | 0.19 | 0.008 | 0.040 | -0.11 | -0.27 | 0.04 | 0.155 | 0.641 |
| New deaths per day (std) | 0.30 | 0.27 | 0.34 | <0.001 | <0.001 | 0.11 | 0.06 | 0.16 | <0.001 | <0.001 | -0.04 | -0.29 | 0.21 | 0.745 | 1.000 |
| **Anxiety symptoms** |  |  |  |  |  |  |  |  |  |  |  |  |  |  |  |
| Stringency index (std) | 0.36 | 0.31 | 0.40 | <0.001 | <0.001 | 0.09 | 0.01 | 0.16 | 0.021 | 0.104 | 0.08 | 0.01 | 0.15 | 0.022 | 0.104 |
| Vaccination (std) | -- | -- | -- | -- | -- | -0.06 | -0.18 | 0.05 | 0.294 | 0.888 | -0.08 | -0.23 | 0.06 | 0.253 | 0.840 |
| New cases per day (std) | 1.24 | 0.35 | 2.13 | 0.006 | 0.040 | 0.12 | 0.05 | 0.19 | 0.001 | 0.011 | 0.10 | -0.02 | 0.22 | 0.116 | 0.482 |
| New deaths per day (std) | 0.08 | 0.05 | 0.11 | <0.001 | <0.001 | 0.05 | 0.02 | 0.09 | 0.002 | 0.017 | 0.17 | -0.07 | 0.41 | 0.163 | 0.602 |

Notes: All predictors were standardised (std) in the total sample to have a mean of 0 and standard deviation of 1, except for the binary variable, COVID-19 infection. ^†^ Number of unique participants, ^‡^ Mean number of time points (week/month) per participant
