# Supplementary material for: Depressive and anxiety symptoms in adults during the COVID-19 pandemic in England: A panel data analysis over 2 years
Source: PLoS Med. 2023 Apr 18;20(4):e1004144. doi: 10.1371/journal.pmed.1004144 (PMC10112796; doi:10.1371/journal.pmed.1004144)
Supplement: S7 Table — (DOCX) [file pmed.1004144.s008.docx]

S7 Table Results from fixed effects models with standardised outcomes across three periods (weighted)

|  | Period I: 1^st^ lockdown  (21/03/2020-23/08/2020)  (N^†^=45,838, T^‡^_mean_=11.5) | | | | | Period II: 2^nd^ & 3^rd^ lockdowns  (21/09/2020-11/04/2021)  (N^†^=26,175, T^‡^_mean_=6.1) | | | | | Period III: freedom  (12/04/2021-14/11/2021)  (N^†^=21,194, T^‡^_mean_=6.3) | | | | |
| --- | --- | --- | --- | --- | --- | --- | --- | --- | --- | --- | --- | --- | --- | --- | --- |
|  | Coef. | 95% CI | | p | q | Coef. | 95% CI | | p | q | Coef. | 95% CI | | p | q |
| **Depressive symptoms** |  |  |  |  |  |  |  |  |  |  |  |  |  |  |  |
| Stringency index (std) | 0.04 | 0.03 | 0.05 | <0.001 | <0.001 | 0.05 | 0.04 | 0.07 | <0.001 | <0.001 | 0.01 | -0.01 | 0.02 | 0.262 | 1.000 |
| Vaccination (std) | -- | -- | -- | -- | -- | 0.07 | 0.04 | 0.09 | <0.001 | <0.001 | -0.02 | -0.04 | 0.01 | 0.271 | 1.000 |
| New cases per day (std) | -0.83 | -1.02 | -0.64 | <0.001 | <0.001 | 0.00 | -0.02 | 0.01 | 0.560 | 1.000 | -0.03 | -0.06 | -0.01 | 0.018 | 0.091 |
| New deaths per day (std) | 0.05 | 0.04 | 0.06 | <0.001 | <0.001 | 0.02 | 0.01 | 0.02 | <0.001 | 0.001 | -0.01 | -0.05 | 0.03 | 0.655 | 1.000 |
| Confidence: government (std) | -0.02 | -0.03 | -0.01 | <0.001 | 0.001 | -0.04 | -0.05 | -0.02 | <0.001 | <0.001 | -0.04 | -0.06 | -0.02 | <0.001 | <0.001 |
| Confidence: healthcare (std) | -0.03 | -0.04 | -0.02 | <0.001 | <0.001 | -0.04 | -0.06 | -0.02 | <0.001 | <0.001 | -0.03 | -0.04 | -0.02 | <0.001 | <0.001 |
| Confidence: essential (std) | -0.04 | -0.05 | -0.03 | <0.001 | <0.001 | -0.02 | -0.03 | -0.01 | 0.002 | 0.012 | -0.01 | -0.02 | 0.00 | 0.139 | 0.668 |
| COVID-19 knowledge (std) | -0.02 | -0.03 | -0.01 | <0.001 | <0.001 | -0.02 | -0.03 | 0.00 | 0.017 | 0.086 | -0.01 | -0.02 | 0.01 | 0.501 | 1.000 |
| COVID-19 stress (std) | 0.07 | 0.06 | 0.07 | <0.001 | <0.001 | 0.03 | 0.02 | 0.05 | <0.001 | <0.001 | 0.02 | 0.01 | 0.03 | 0.007 | 0.038 |
| COVID-19 infection | 0.06 | 0.03 | 0.09 | <0.001 | <0.001 | 0.10 | 0.06 | 0.15 | <0.001 | <0.001 | 0.19 | 0.13 | 0.25 | <0.001 | <0.001 |
| Social support (std) | -0.16 | -0.18 | -0.15 | <0.001 | <0.001 | -0.17 | -0.19 | -0.14 | <0.001 | <0.001 | -0.18 | -0.20 | -0.15 | <0.001 | <0.001 |
| **Anxiety symptoms** |  |  |  |  |  |  |  |  |  |  |  |  |  |  |  |
| Stringency index (std) | 0.03 | 0.03 | 0.04 | <0.001 | <0.001 | 0.03 | 0.01 | 0.04 | 0.001 | 0.005 | 0.02 | 0.01 | 0.03 | 0.03 | 0.035 |
| Vaccination (std) | -- | -- | -- | -- | -- | 0.03 | 0.01 | 0.06 | 0.011 | 0.078 | -0.02 | -0.05 | 0.00 | 0.00 | 0.519 |
| New cases per day (std) | 0.07 | -0.11 | 0.26 | 0.422 | 1.000 | 0.00 | -0.02 | 0.01 | 0.799 | 1.000 | 0.01 | -0.02 | 0.03 | 0.07 | 1.000 |
| New deaths per day (std) | 0.01 | 0.01 | 0.02 | <0.001 | <0.001 | 0.01 | 0.00 | 0.01 | 0.030 | 0.184 | 0.03 | -0.02 | 0.08 | 0.01 | 0.961 |
| Confidence: government (std) | 0.00 | 0.00 | 0.01 | 0.359 | 1.000 | -0.03 | -0.05 | -0.01 | <0.001 | 0.002 | -0.03 | -0.05 | -0.01 | 0.00 | 0.032 |
| Confidence: healthcare (std) | -0.03 | -0.03 | -0.02 | <0.001 | <0.001 | -0.04 | -0.05 | -0.02 | <0.001 | <0.001 | -0.03 | -0.05 | -0.02 | -0.03 | <0.001 |
| Confidence: essential (std) | -0.06 | -0.06 | -0.05 | <0.001 | <0.001 | -0.02 | -0.03 | -0.01 | <0.001 | 0.003 | -0.01 | -0.03 | 0.00 | -0.06 | 0.209 |
| COVID-19 knowledge (std) | -0.02 | -0.03 | -0.01 | <0.001 | <0.001 | -0.02 | -0.03 | 0.00 | 0.013 | 0.087 | 0.00 | -0.01 | 0.02 | -0.02 | 1.000 |
| COVID-19 stress (std) | 0.11 | 0.10 | 0.11 | <0.001 | <0.001 | 0.05 | 0.04 | 0.07 | <0.001 | <0.001 | 0.04 | 0.02 | 0.05 | 0.11 | <0.001 |
| COVID-19 infection | 0.01 | -0.02 | 0.03 | 0.645 | 1.000 | 0.04 | 0.00 | 0.07 | 0.044 | 0.247 | 0.05 | 0.00 | 0.10 | 0.01 | 0.315 |
| Social support (std) | -0.12 | -0.13 | -0.11 | <0.001 | <0.001 | -0.13 | -0.15 | -0.11 | <0.001 | <0.001 | -0.13 | -0.16 | -0.11 | -0.12 | <0.001 |

Notes: All predictors and outcomes were standardised (std) in the total sample to have a mean of 0 and standard deviation of 1, except for the binary variable, COVID-19 infection. ^†^ Number of unique participants, ^‡^ Mean number of time points (week/month) per participant
