# Supplementary material for: Depressive and anxiety symptoms in adults during the COVID-19 pandemic in England: A panel data analysis over 2 years
Source: PLoS Med. 2023 Apr 18;20(4):e1004144. doi: 10.1371/journal.pmed.1004144 (PMC10112796; doi:10.1371/journal.pmed.1004144)
Supplement: S8 Table — (DOCX) [file pmed.1004144.s009.docx]

S8 Table Results from fixed effects models comparing Period II models with and without seasonal dummy (weighted)

|  | Period II: 2^nd^ & 3^rd^ lockdowns  (21/09/2020-11/04/2021)  (N^†^=26,175, T^‡^­_mean_=6.1) | | | | | Period II: 2^nd^ & 3^rd^ lockdowns  (21/09/2020-11/04/2021)  (N^†^=26,175, T^‡^_mean_=6.1) | | | | |
| --- | --- | --- | --- | --- | --- | --- | --- | --- | --- | --- |
|  | Coef. | 95% CI | | p | q | Coef. | 95% CI | | p | q |
| **Depressive symptoms** |  |  |  |  |  |  |  |  |  |  |
| Stringency index (std) | 0.30 | 0.21 | 0.39 | <0.001 | <0.001 | 0.34 | 0.22 | 0.46 | <0.001 | <0.001 |
| Vaccination (std) | 0.38 | 0.22 | 0.53 | <0.001 | <0.001 | 0.38 | 0.23 | 0.54 | <0.001 | <0.001 |
| New cases per day (std) | -0.03 | -0.12 | 0.06 | 0.560 | 1.000 | -0.01 | -0.11 | 0.09 | 0.891 | 1.000 |
| New deaths per day (std) | 0.09 | 0.05 | 0.13 | <0.001 | 0.001 | 0.09 | 0.04 | 0.13 | <0.001 | <0.001 |
| Confidence: government (std) | -0.20 | -0.29 | -0.11 | <0.001 | <0.001 | -0.20 | -0.29 | -0.11 | <0.001 | <0.001 |
| Confidence: healthcare (std) | -0.22 | -0.31 | -0.12 | <0.001 | <0.001 | -0.22 | -0.31 | -0.12 | <0.001 | <0.001 |
| Confidence: essential (std) | -0.11 | -0.18 | -0.04 | 0.002 | 0.012 | -0.11 | -0.18 | -0.04 | 0.002 | 0.008 |
| COVID-19 knowledge (std) | -0.09 | -0.16 | -0.02 | 0.017 | 0.086 | -0.09 | -0.16 | -0.02 | 0.016 | 0.060 |
| COVID-19 stress (std) | 0.19 | 0.13 | 0.25 | <0.001 | <0.001 | 0.19 | 0.13 | 0.25 | <0.001 | <0.001 |
| COVID-19 infection | 0.58 | 0.33 | 0.83 | <0.001 | <0.001 | 0.58 | 0.33 | 0.83 | <0.001 | <0.001 |
| Social support (std) | -0.95 | -1.09 | -0.80 | <0.001 | <0.001 | -0.95 | -1.09 | -0.80 | <0.001 | <0.001 |
| Winter (21/12/20-19/03/21)* |  |  |  |  |  | -0.06 | -0.15 | 0.03 | 0.228 | 0.772 |
| **Anxiety symptoms** |  |  |  |  |  |  |  |  |  |  |
| Stringency index (std) | 0.13 | 0.06 | 0.21 | 0.001 | 0.005 | 0.22 | 0.12 | 0.32 | <0.001 | <0.001 |
| Vaccination (std) | 0.16 | 0.04 | 0.28 | 0.011 | 0.078 | 0.17 | 0.05 | 0.29 | 0.007 | 0.033 |
| New cases per day (std) | -0.01 | -0.09 | 0.07 | 0.799 | 1.000 | 0.03 | -0.05 | 0.11 | 0.479 | 1.000 |
| New deaths per day (std) | 0.04 | 0.00 | 0.07 | 0.030 | 0.184 | 0.03 | 0.00 | 0.07 | 0.044 | 0.152 |
| Confidence: government (std) | -0.16 | -0.24 | -0.07 | <0.001 | 0.002 | -0.16 | -0.24 | -0.07 | <0.001 | <0.001 |
| Confidence: healthcare (std) | -0.18 | -0.24 | -0.11 | <0.001 | <0.001 | -0.18 | -0.24 | -0.11 | <0.001 | <0.001 |
| Confidence: essential (std) | -0.11 | -0.17 | -0.05 | <0.001 | 0.003 | -0.11 | -0.17 | -0.05 | <0.001 | <0.001 |
| COVID-19 knowledge (std) | -0.08 | -0.14 | -0.02 | 0.013 | 0.087 | -0.08 | -0.14 | -0.02 | 0.013 | 0.054 |
| COVID-19 stress (std) | 0.27 | 0.21 | 0.33 | <0.001 | <0.001 | 0.27 | 0.21 | 0.33 | <0.001 | <0.001 |
| COVID-19 infection | 0.18 | 0.00 | 0.35 | 0.044 | 0.247 | 0.18 | 0.00 | 0.35 | 0.045 | 0.152 |
| Social support (std) | -0.66 | -0.76 | -0.55 | <0.001 | <0.001 | -0.65 | -0.76 | -0.55 | <0.001 | <0.001 |
| Winter (21/12/20-19/03/21)* |  |  |  |  |  | -0.11 | -0.19 | -0.03 | 0.005 | 0.027 |

Notes: All predictors and outcomes were standardised (std) in the total sample to have a mean of 0 and standard deviation of 1, except for the binary variable, COVID-19 infection. ^†^ Number of unique participants, ^‡^ Mean number of time points (week/month) per participant, * winter dates obtained from Met Office
