# Supplementary material for: Depressive and anxiety symptoms in adults during the COVID-19 pandemic in England: A panel data analysis over 2 years
Source: PLoS Med. 2023 Apr 18;20(4):e1004144. doi: 10.1371/journal.pmed.1004144 (PMC10112796; doi:10.1371/journal.pmed.1004144)
Supplement: S1 Fig — (DOCX) [file pmed.1004144.s010.docx]

No follow-up

(N= 2,459)

No follow-up

(N= 6,546)

Total number of participants (consented)

(N= 73,222)

Participants living in England

(N= 59,810)

Missing outcome/weighting/date variables

(N= 2,044)

Participants in Wales (N= 7,727), Scotland (N= 4,812) & Northern Ireland (N= 873)

**Descriptive analysis sample**

**(N=57,766, T_mean_=16.4)**

Total participants in Period II

(N= 32,721)

**Final sample II (21/09/2020-11/04/2021)**

**(N= 26,175, T_mean_=6.1, T_median_=7)**

Total participants in Period I

(N= 57,452)

**Final sample I (21/03/2020-23/08/2020)**

**(N= 45,838, T_mean_=11.5, T_median_=12)**

Total participants in Period III

(N= 23,653)

**Final sample III (12/04/2021-14/11/2021)**

**(N= 21,194, T_mean_=6.3, T_median_=7)**

No follow-up

(N= 11,614)

Main analysis sample: fixed effects (N=57,692)

Missing predictors

(N=74)

S1 Fig. Sample selection diagram (N=number of unique participants, T=number of time points)
