# Supplementary material for: Depressive and anxiety symptoms in adults during the COVID-19 pandemic in England: A panel data analysis over 2 years
Source: PLoS Med. 2023 Apr 18;20(4):e1004144. doi: 10.1371/journal.pmed.1004144 (PMC10112796; doi:10.1371/journal.pmed.1004144)

S2 Fig. Daily changes in COVID-19 policy responses (stringency index) in England from January 2020 to April 2022


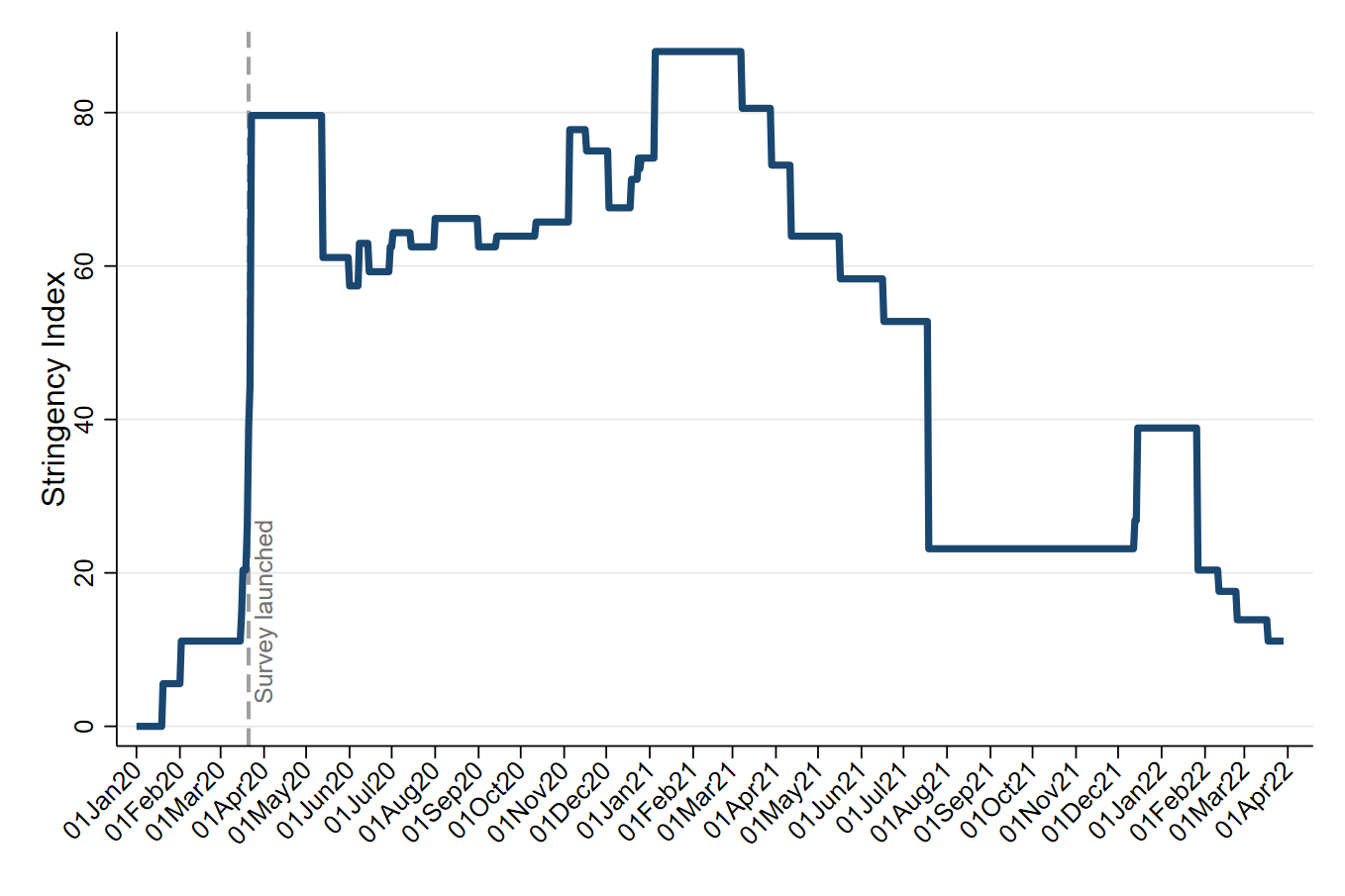

Supplement: S2 Fig — (DOCX) [file pmed.1004144.s011.docx]
