# Supplementary material for: Depressive and anxiety symptoms in adults during the COVID-19 pandemic in England: A panel data analysis over 2 years
Source: PLoS Med. 2023 Apr 18;20(4):e1004144. doi: 10.1371/journal.pmed.1004144 (PMC10112796; doi:10.1371/journal.pmed.1004144)
Supplement: S3 Fig — (DOCX) [file pmed.1004144.s012.docx]

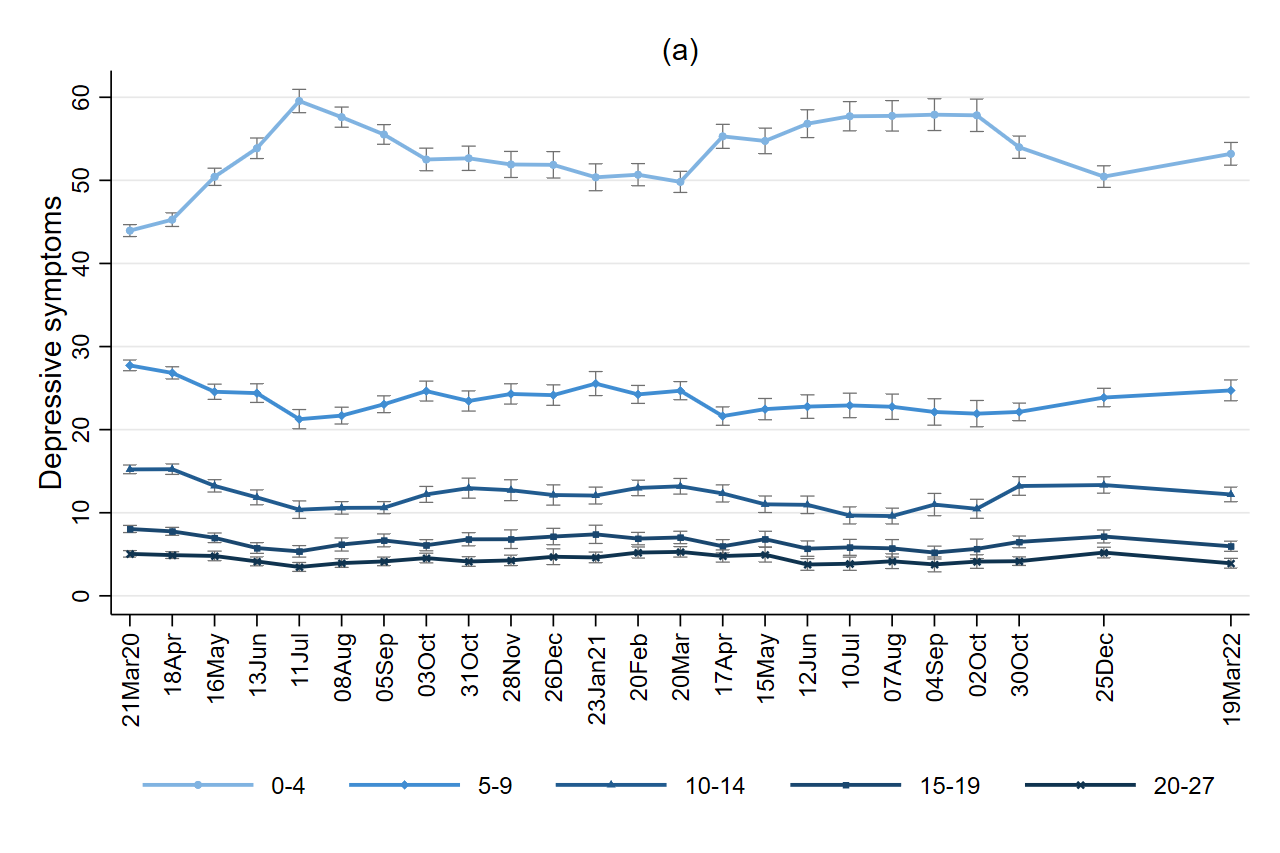

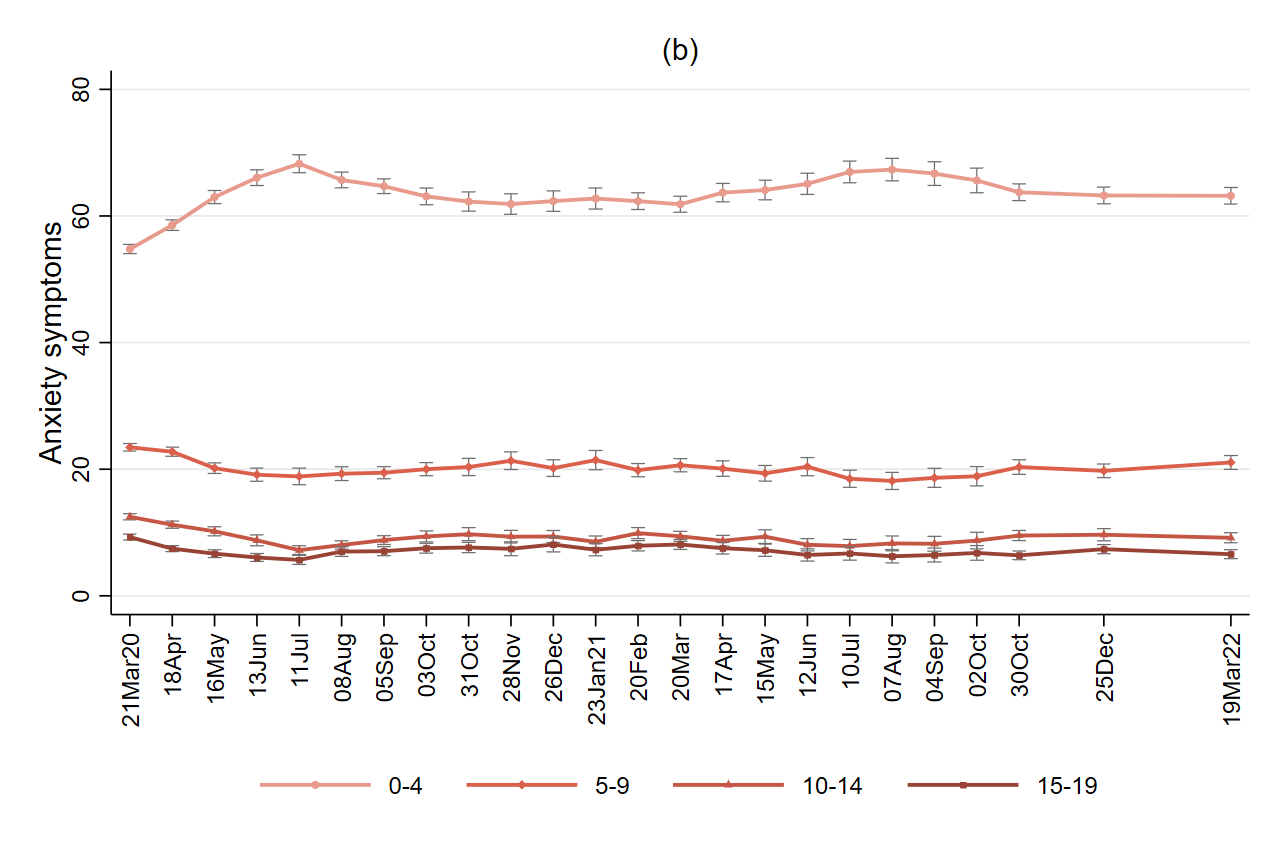


S3 Fig. Trends of depressive (weighted percentages in five categories) and anxiety symptoms (weighted percentages in four categories) over time from March 2020 to March 2022
