# Supplementary figures and images for: Depressive and anxiety symptoms in adults during the COVID-19 pandemic in England: A panel data analysis over 2 years
Source: PLoS Med. 2023 Apr 18;20(4):e1004144. doi: 10.1371/journal.pmed.1004144 (PMC10112796; doi:10.1371/journal.pmed.1004144)

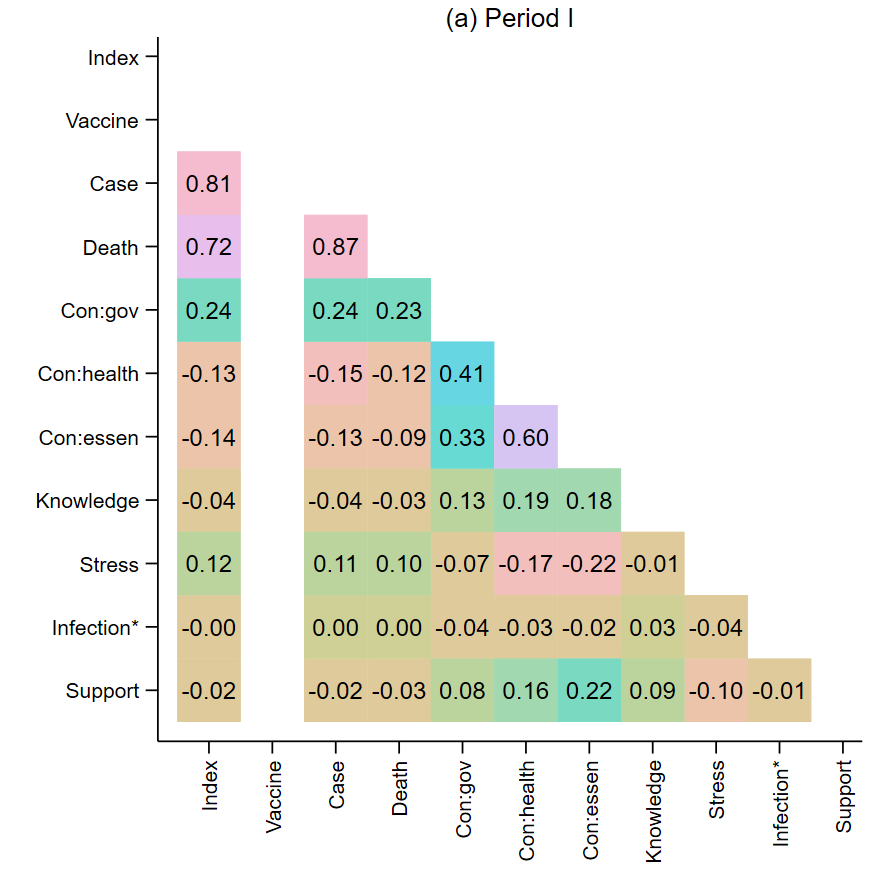

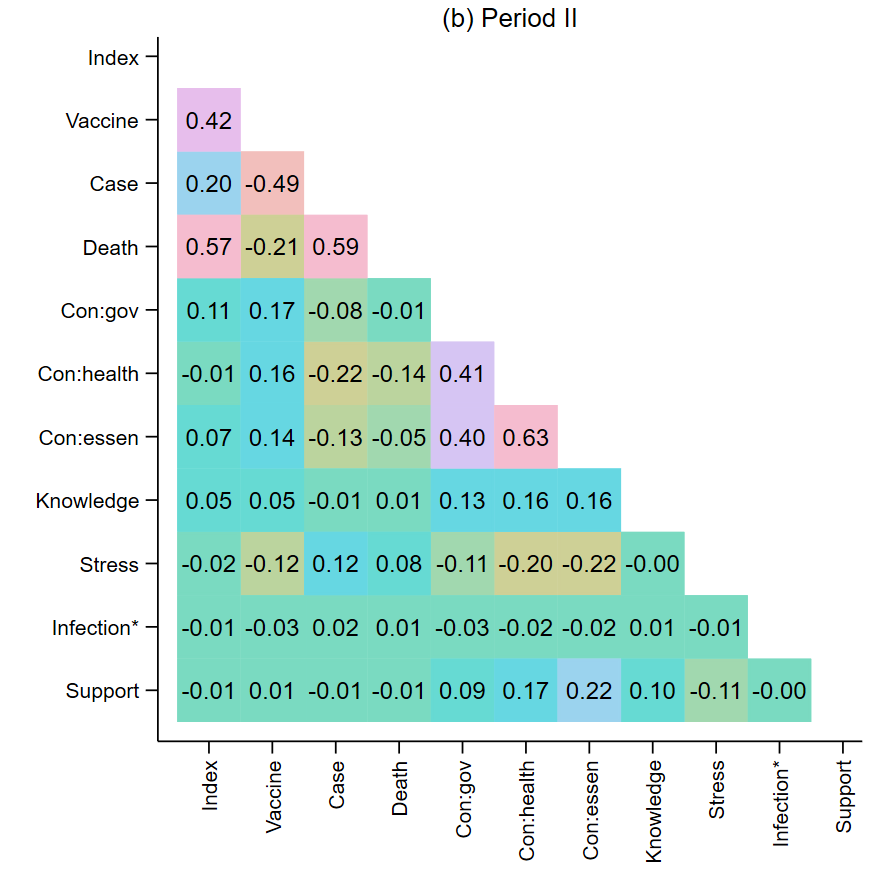


S4 Fig. Correlation matrix of predictors across study periods (unweighted)

Note: * binary variable


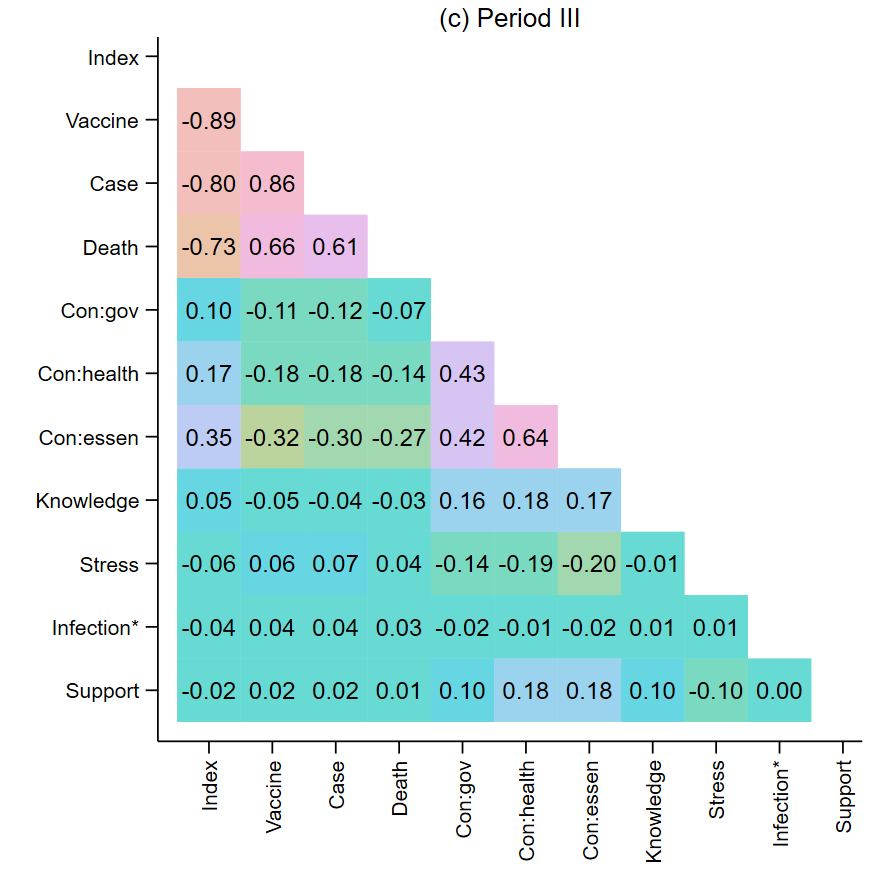

Supplement: S4 Fig — (DOCX) [file pmed.1004144.s013.docx]
